# Hidden Resistances: How Routine Whole-Genome Sequencing Uncovered an Otherwise Undetected *bla*_NDM-1_ Gene in Vibrio alginolyticus from Imported Seafood

**DOI:** 10.1128/spectrum.04176-22

**Published:** 2023-01-05

**Authors:** Jacqueline M. Morris, Karolina Mercoulia, Mary Valcanis, Claire L. Gorrie, Norelle L. Sherry, Benjamin P. Howden

**Affiliations:** a Department of Microbiology and Immunology, University of Melbourne at the Peter Doherty Institute for Infection and Immunity, Melbourne, Victoria, Australia; b Microbiological Diagnostic Unit Public Health Laboratory, University of Melbourne at the Peter Doherty Institute for Infection and Immunity, Melbourne, Victoria, Australia; University at Albany, State University of New York

**Keywords:** antimicrobial resistance, NDM-1, *Vibrio alginolyticus*, whole-genome sequencing, prawn/shrimp, genomics, seafood

## Abstract

Vibrio alginolyticus causes vibriosis of marine vertebrates, invertebrates, and humans, and while there have been several reports of multidrug resistance in V. alginolyticus, carbapenem resistance is rare. V. alginolyticus strain AUSMDU00064140 was isolated in Melbourne, Australia, from imported prawns. Routine genomic surveillance detected the presence of a full-length *bla*_NDM-1_ gene, subsequently shown to be collocated with additional acquired antimicrobial resistance genes on a resistance cassette on the largest chromosome, flanked by mobilization gene annotations. Comparisons to a previously described V. alginolyticus plasmid, pC1349, revealed differing gene content and arrangements between the resistance cassettes. Phylogenetic analysis was performed against a local and global data set (*n* = 109), demonstrating that AUSMDU00064140 was distinct and did not cluster with any other strains. Despite the presence of the complete *bla*_NDM-1_ gene and positive phenotypic assays for carbapenemase production, carbapenem MICs were low (meropenem MIC ≤0.5 mg/liter). However, it is still possible that this gene may be transferred to another species in the environment or a host, causing phenotypic carbapenem resistance and presenting a risk of great public health concern.

**IMPORTANCE** Carbapenems are last-line antimicrobials, vital for use in human medicine. Antimicrobial resistance determinants such as *bla*_NDM_ (New Delhi metallo-β-lactamase producing) genes conferring resistance to the carbapenem class of antimicrobials, are typically found in *Enterobacterales* (first described in 2009 from a Klebsiella pneumoniae isolate). Our study shows that Vibrio alginolyticus isolated from cooked prawn is able to harbor antimicrobial resistance (AMR) genes of public health concern, specifically a chromosomally located *bla*_NDM-1_ gene, and there is the potential for transmission of resistance genes. This may be linked with antimicrobial use in low- and middle-income settings, which has typically been high, unregulated, or not reported. Many countries, including Thailand, have implemented national strategic plans to incorporate the World Health Organization (WHO)’s Global Action Plan (2015) recommendations of a global One Health approach, including increased resources for surveillance of antimicrobial usage and AMR; however, efficient antimicrobial surveillance systems incorporating genomic and phenotypic testing of isolates are still lacking in many jurisdictions.

## OBSERVATION

Vibrio alginolyticus is a zoonotic Gram-negative bacterium, typically found worldwide in aquaculture, marine, estuarine, and coastal environments. V. alginolyticus causes vibriosis of marine vertebrates and invertebrates (fish, mollusks, and crustaceans), resulting in economic losses to the aquaculture and mariculture industries (1). In humans, V. alginolyticus is implicated in wound infections, ocular and intracranial infections, myringitis, and otitis primarily as a result of exposure to the pathogen in water (2). Ingestion of contaminated foods may also cause vibriosis and gastrointestinal infections. Symptoms can range from mild to severe watery diarrhea, abdominal pain, nausea, vomiting, and fevers, generally lasting 2 to 5 days (3, 4). Vibrio spp. are generally susceptible to antimicrobials. However, there have been several reports of resistance to multiple antimicrobial classes such as ampicillin, penicillin, and tetracycline (5–7). In addition, a recent study identified the presence of a plasmid carrying the *bla*_NDM-1_ gene in V. alginolyticus, encoding resistance to the last-line β-lactam antimicrobial class, carbapenems (5–7). Since carbapenems have been listed as critically important for human medicine (8), resistance genes have been described predominately in *Enterobacterales* such as Klebsiella pneumoniae and Escherichia coli (9). Here, we describe a similar finding in a V. alginolyticus isolated from imported seafood in Australia, highlighting the potential risks of imported antimicrobial resistance (AMR) in seafoods and the value of genomic surveillance.

### Routine whole-genome sequencing.

V. alginolyticus strain AUSMDU00064140, together with Vibrio fluvialis, was isolated in Melbourne, Australia, from cooked prawns imported from Thailand in 2021 and referred to the Microbiological Diagnostic Unit Public Health Laboratory (MDU PHL) for identification and further characterization. Routine whole-genome sequencing (WGS) was performed on the Illumina NextSeq500/550 platforms and subsequent screening of AMR genes found that no resistance genes were detected in the V. fluvialis strain. However, the presence of a *bla*_NDM-1_ gene was uncovered in V. alginolyticus AUSMDU00064140, requiring further investigation.

### Genomic characterization and comparisons to global isolates.

V. alginolyticus AUSMDU00064140 was compared to a global V. alginolyticus data set of complete and draft genomes to provide genetic context (*n* = 109; supplemental material for detailed methods and Table S1 for isolates list). Draft genomes for all Illumina data were assembled using Shovill (version 1.0.9, https://github.com/tseemann/shovill) with SKESA assembler (version 2.4.0) (10). Long read sequencing was also performed for V. alginolyticus AUSMDU00064140 on the Oxford Nanopore GridION X5 platform (with FLO-MIN106D R9 flow cells). A complete genome for V. alginolyticus AUSMDU00064140 (GCF_026639325.1), using both short and long reads, was assembled with Unicycler (version 0.4.8) (11).

**FIG 1:**
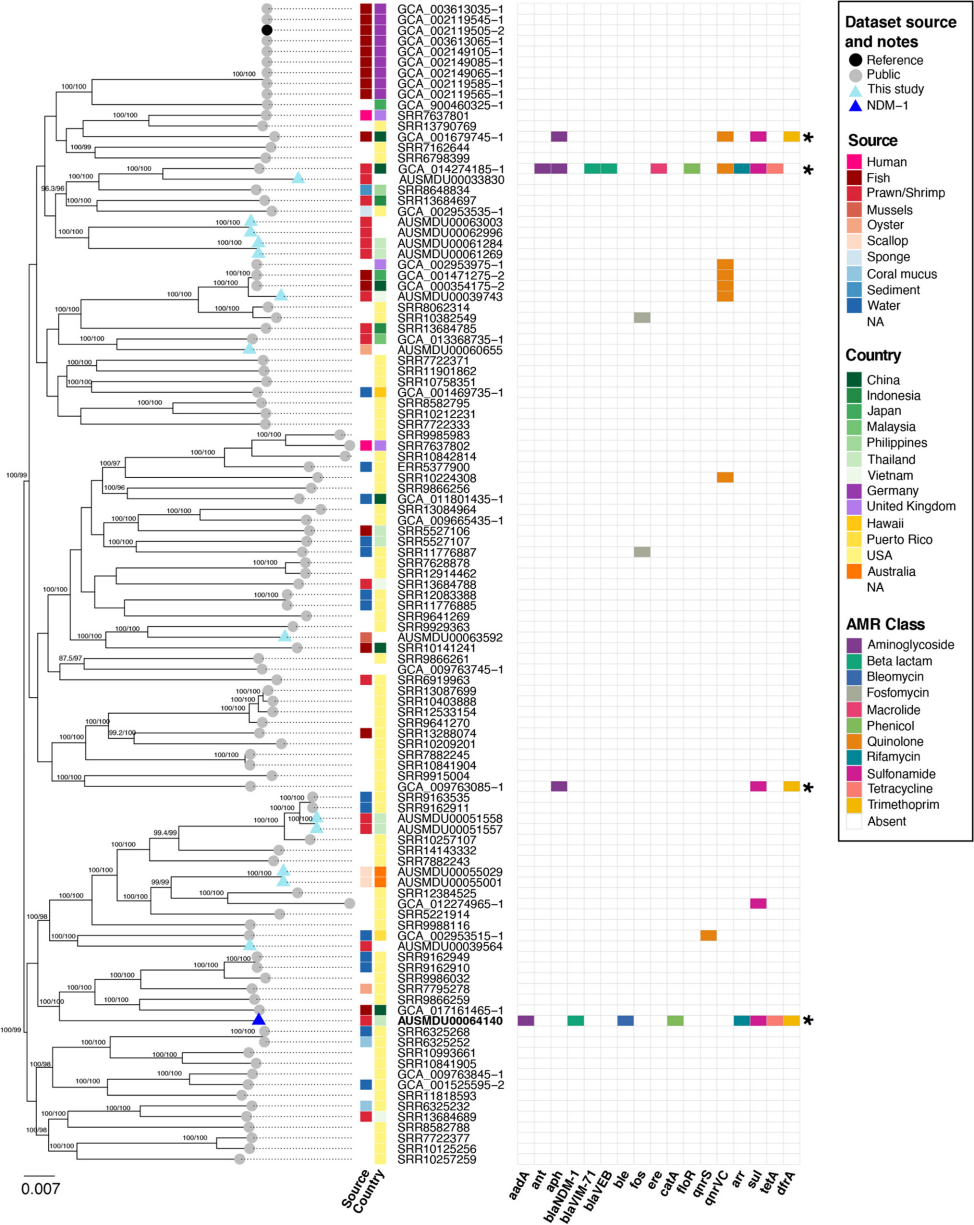
Midpoint-rooted maximum-likelihood phylogeny and AMR gene presence/absence for V. alginolyticus isolates. The phylogenetic tree was inferred from 189,922 core SNP sites; values for all trusted branches (SH-aLRT ≥ 80% and UFboot ≥ 95%) are shown; the tree scale bar indicates the number of substitutions per site. The tree tips highlight isolates novel to this study (light blue triangles), including AUSMDU00064140 (dark blue triangle), and isolates from public repositories (gray circles) including the selected reference, GCA_002119505.2_K01M1 (black circles). Each isolate’s source and country of isolation are shown according to the legend; note that the country of isolation does not necessarily represent the origin of the sample. The presence or absence of acquired AMR genes is visualized as a heat map, colored by drug class according to the legend; asterisks denote multidrug resistance (resistant to ≥3 drug classes). No genomic data were publicly available for comparison of the chromosomes of V. alginolyticus strain Vb1394, associated with the previously described *bla*_NDM-1_ containing-plasmid pC1394 (MH457126.1).

V. alginolyticus AUSMDU00064140 belongs to a novel sequence type, ST212 (as assigned by PubMLST [12, 13] following the submission of the novel allele combination) and consists of two chromosomes (CP110670, 3.4 Mb and CP110671, 1.8 Mb in size) and a plasmid (CP110672, 51 kb). Single-nucleotide polymorphisms (SNPs) were called by aligning all genomes to the reference V. alginolyticus K01M1 (GCA_002119505.2) (14) using Snippy (version 4.6.0 https://github.com/tseemann/snippy; mincov 10, minfrac 0.9). The core SNP alignment (189,922 bp) was used with IQ-TREE (version 2.1.4; using constant sites, 1,000 SH-like approximate likelihood ratio tests (SH-aLRT) and ultrafast (UF) bootstraps and GTR+F+G4 model of evolution) to infer the maximum-likelihood phylogenetic tree, in which V. alginolyticus AUSMDU00064140 is phylogenetically distinct from all other isolates. V. alginolyticus AUSMDU00064140 is most closely related to five isolates sourced from water, fish, or oysters from the United States (SRR9162949, SRR9162910, SRR9986032, SRR7795278, and SRR9866259) and one from fish from China (GCA_017161465-1) (Fig. 1). AMR genes were screened for using the abritAMR tool (version 1.0.2, https://github.com/MDU-PHL/abritamr; based on NCBI AMRFinderPlus) (15, 16). V. alginolyticus AUSMDU00064140 had complete matches (100% coverage and amino acid identity) for *bla*_NDM-1_ and other resistance determinants, including CARB-42 and *tetA(D)*, among others (Fig. 1; Tables S1 and S2). In the wider V. alginolyticus data set (*n* = 108), all isolates encoded either CARB-42 or CARB-56, in addition to *tet(34)* and *tet(35)*; these genes were considered intrinsic (Table S1). A small number of isolates had additional resistance genes detected, considered to be acquired genes, as shown in Fig. 1. Multidrug resistance (resistant to three or more drug classes) was detected in four V. alginolyticus isolates: AUSMDU00064140 (eight classes); GCA_014274185.1, strain Vb1833 (eight classes, isolated from shrimp in China); GCA_001679745.1, strain ZJ-T (four classes, isolated from fish in China); and GCA_009763085.1, strain 2014V-1011 (three classes, isolated in USA).

**FIG 2:**
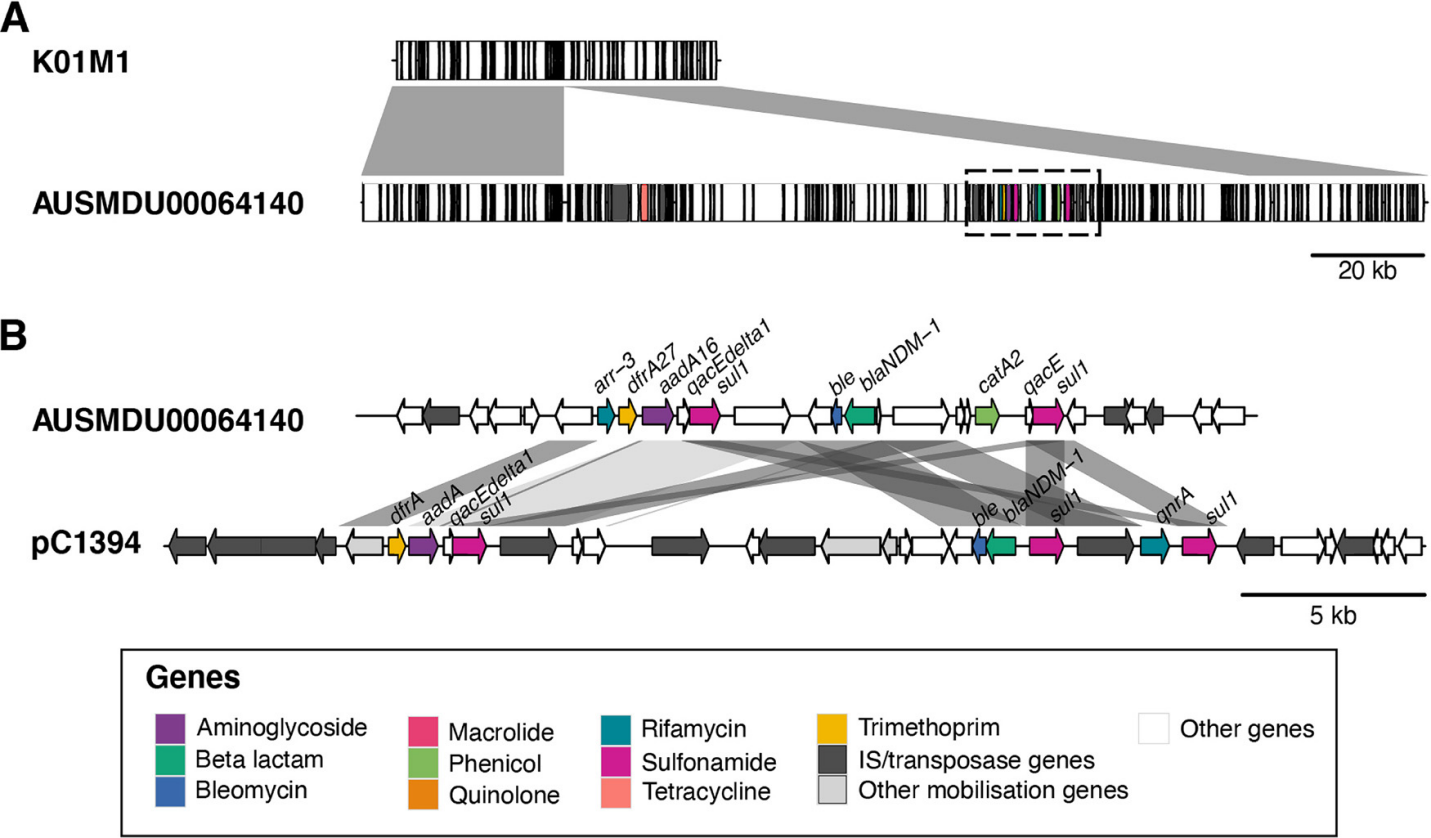
The location, structure, and nucleotide similarity of V. alginolyticus strain AUSMDU00064140 AMR cassette and surrounding genes. (A) Comparison of the 2.72 Mb to 2.78 Mb region (shown in reverse orientation) of V. alginolyticus strain K01M1 chromosome (GCA_002119505.2) and the 665 kb to 860 kb region of AUSMDU00064140 chromosome encoding the *bla_NDM-1_* gene. The genes are represented by rectangles and colored according to the legend. The gray connecting blocks indicate > 98% nucleotide identity between the two sequences, and the black dotted box indicates the 25 kb region of AUSMDU00064140 shown in panel B. (B) Comparison of AUSMDU00064140 (775 kb to 800 kb region) and the NDM-1 containing-plasmid pC1394 (MH457126.1, V. alginolyticus strain Vb1394) (100 kb to 130 kb region). Genes and their orientation are indicated by arrows and colored according to the legend; the gray connecting blocks indicate regions of >95% nucleotide identity.

Nucleotide BLAST comparisons of V. alginolyticus AUSMDU00064140 to the reference V. alginolyticus K01M1 (GCA_002119505.2) indicate an insertion into the largest chromosome of V. alginolyticus AUSMDU00064140, which includes all acquired AMR genes (Fig. 2A). In addition, comparisons of V. alginolyticus AUSMDU00064140 to the NDM-1 containing-plasmid pC1394 (MH457126.1, V. alginolyticus strain Vb1394) (6) showed similarity to the resistance cassette encoded on pC1394, including 100% coverage and nucleotide identity of the *bla*_NDM-1_ gene (Fig. 2B), but with differences in the gene arrangement and content in this region. No AMR genes were detected on the V. alginolyticus AUSMDU00064140 plasmid (pAUSMDU00064140), which appears to be a novel plasmid (supplemental material).

### Antimicrobial resistance phenotyping and significance.

Antimicrobial susceptibility was tested using the Sensititre broth microdilution system (ThermoFisher Scientific), and phenotypic carbapenemase detection was performed using the carbapenemase inactivation method (CIM) test, as previously described (17). V. alginolyticus AUSMDU00064140 was CIM positive (i.e., phenotypic carbapenemase activity detected) and yet exhibited low imipenem and meropenem MICs of ≤1 and ≤0.5 mg/liter, respectively (Table 1). Carbapenem susceptibility is unusual among NDM-producing isolates but has been previously described in Vibrio spp. (6) and Acinetobacter spp. (18). It is hypothesized that altered phenotypic profiles may be due to differential gene expression in various species, as conjugation experiments demonstrated transferable carbapenem resistance when plasmids were transformed into an azide-resistant E. coli J53 strain, resulting in significant resistance to meropenem in over 50% of the transconjugants (6, 18). Hence, the presence of *bla_NDM-1_* in V. alginolyticus is concerning due to the potential for gene transmission in host and environment (1, 7, 19, 20). Significantly, phenotypic testing (carbapenem MIC) alone would not have detected a carbapenemase in this isolate, demonstrating the additional value of genomics in detecting AMR determinants of public health significance.

**TABLE 1:** MICs of V. alginolyticus strain AUSMDU00064140^*a*^

Class	Antibiotic	MIC (mg/liter)
Penicillins	Ampicillin	>16
	Ampicillin-sulbactam	>16
Cephalosporins	Cefazolin	>16
	Cefepime	16
	Ceftazidime-avibactam	≤2
	Ceftazidime	>16
	Ceftriaxone	>32
Quinolones	Ciprofloxacin	≤0.5
Carbapenems	Doripenem	≤0.5
	Ertapenem	≤0.25
	Imipenem	≤1
	Meropenem	≤0.5
Aminoglycosides	Gentamicin	≤2
	Tobramycin	≤2
Tetracyclines	Tetracycline	8
Sulfonamides	Sulfamethoxazole-trimethoprim	≤2

a No clinical breakpoints available. Carbapenem susceptibility extrapolated from *Enterobacterales* breakpoints (European Committee on Antimicrobial Susceptibility Testing [EUCAST]).

### Further discussion.

Globally, the majority of public health surveillance programs do not actively test and analyze non-cholera Vibrio spp. (7, 20, 21). Imported food entering Australia is inspected and controlled as per the Imported Food Inspection Scheme with only V. cholerae considered a risk in imported ready-to-eat cooked prawns and shrimp. Although not targeted, V. alginolyticus and V. fluvialis resemble V. cholerae on selective media and therefore are, on occasion, referred for further identification.

As a result, foodborne illness due to noncholera Vibrio spp. may go undetected. This, coupled with the fact that vibriosis is not notifiable in humans, presents a gap in the estimating the burden of disease attributed to Vibrio spp. including V. alginolyticus. Cooking time for ready-to-eat prawns and shrimp is quick, and often prawns are not uniformly cooked if densely packed during the boiling process. This, along with potential cross contamination during the production process, allows viable strains of Vibrio spp. to remain in the final product. With increasing reports on AMR in Vibrio spp., this gap may result in multidrug-resistant Vibrio spp. going undetected in our food, particularly imported foods (1, 7, 20, 22).

Our study shows that V. alginolyticus isolated from cooked prawn can harbor AMR genes of public health concern, with previous studies demonstrating the potential for transmission of resistance genes. We believe there is a need for non-cholera Vibrio spp. to be included in surveillance activities, particularly when screening seafood from aquaculture systems, which are a vital industry for food security globally and protein production and yet represent a potential AMR hot spot (23–25).

### Data availability.

Whole-genome sequence reads from V. alginolyticus isolates sequenced in this study and the assembled genome for V. alginolyticus AUSMDU00064140 (GCF_026639325.1) are deposited in the National Centre for Biotechnology Information (NCBI) Sequence Read Archive (SRA) and Genome under the BioProject PRJNA856407 and the accession numbers listed in Table S1.

## Supplementary Material

Reviewer comments
