# Supplementary material for: Hidden Resistances: How Routine Whole-Genome Sequencing Uncovered an Otherwise Undetected blaNDM-1 Gene in Vibrio alginolyticus from Imported Seafood
Source: Microbiol Spectr. 2023 Jan 5;11(1):e04176-22. doi: 10.1128/spectrum.04176-22 (PMC9927303; doi:10.1128/spectrum.04176-22)
Supplement: Supplemental file 1 — Text S1. Download spectrum.04176-22-s0001.pdf, PDF file, 0.10 MB [file spectrum.04176-22-s0001.pdf]

Supplementary Information

Dataset collection, sequencing, and quality control

In addition to AUSMDU00064140, 13 recent *Vibrio alginolyticus* isolates received between August 2019 to July 2021, were processed for whole genome sequencing. Genomic DNA was extracted using QIAsymphony DSP Virus/Pathogen Mini Kit (Qiagen) using an in-house lysis buffer and 150 bp (base pairs) paired-end multiplex libraries were prepared using Nextera XT DNA Library preparation kit (Illumina) and sequenced using the Illumina NextSeq500/550 platform. Twenty-eight *V. alginolyticus* genome assemblies were collected from RefSeq, in addition to 134 publicly available Illumina paired-end short read data sets, available from 20th August, 2021. Species identification was determined using kraken2 through the bohra pipeline (v2.0.0, https://github.com/kristyhoran/bohra) and average nucleotide identity (ANI) for all isolates was performed using pyani (v 0.2.11, https://github.com/widdowquinn/pyani). Isolates that were classed as *V. alginolyticus* and had an ANI ≥ 97% to the National Centre for Biotechnology Information (NCBI) representative genome, *V. alginolyticus* strain FDAARGOS_97 (GCA_001471275-2) were used in this study. The final dataset was refined to 14 locally collected and sequenced genomes, 27 RefSeq genome assemblies and 99 Illumina paired-end short read datasets. All isolates and corresponding metadata are included in Supplementary Table 1. Adapters were removed and reads were filtered to above 75 bp in length and quality scores above 30 using Trim Galore (v0.6.7 https://github.com/FelixKrueger/TrimGalore/tree/0.6.7) employing cutadapt (v1.18) (Martin, 2011) and FastQC (v0.11.9 https://www.bioinformatics.babraham.ac.uk/projects/fastqc/).

DNA was extracted for long read sequencing of AUSMDU00064140 using the GenElute Bacterial Genomic DNA kit (Sigma- Aldrich), followed by long read library preparation with

the native DNA barcoding and Ligation Sequencing Kit (SQK-LSK 109, Oxford Nanopore Technologies, ONT). Long-read genomic data was sequenced on the Oxford Nanopore GridION X5 platform (with FLO- MIN106D R9 flow cells) and basecalled using Guppy (v5.0.16) with the high accuracy protocol. Adapters were removed, reads spit and demultiplexing conducted using Porechop (v0.2.4, https://github.com/rrwick/Porechop) employing stringent binning with the flags (--barcode_threshold 85 and --require_two_barcodes), followed by quality filtering using Filtlong (v 0.2.1, https://github.com/rrwick/Filtlong) (--min_length 1000 --target_bases 540000000 --keep_percent 90).

Genome assembly, annotation and sequence typing

De novo draft assemblies for all Illumina datasets were performed using Shovill (v1.0.9 https://github.com/tseemann/shovill) with SKESA assembler (v2.4.0 https://genomebiology.biomedcentral.com/articles/10.1186/s13059-018-1540-z).
A complete genome for AUSMDU00064140 was assembled using both ONT long reads and Illumina short reads with Unicycler (v0.4.8) [1].
Annotation of all genome assemblies was performed using Prokka (v1.14.6 https://github.com/tseemann/prokka), with *V. alginolyticus* strain FDAARGOS_97 (GCA_001471275-2) proteins for initial annotations. Genome statistics were collated from Prokka outputs and using Nullarbor-fa v2.0 (https://github.com/tseemann/nullarbor). *In silico* multi-locus sequence typing was performed by MLST (v2.16 https://github.com/tseemann/mlst) with BLAST+ (v2.10.0) (Altschul et al., 1990). The novel MLST allele combination detected in AUSMDU00064140 was submitted to PubMLST for sequence type assignment (https://pubmlst.org/bigsdb?db=pubmlst_vibrio_seqdef).

Antimicrobial resistance (AMR) gene profiles

All genomes were screened for AMR genes using abriTAMR (v1.0.2 https://github.com/MDU-PHL/abritamr) against the AMRFinderPlus database (v 2021-09-30.1) [2, 3].

Phylogeny

Constant sites for phylogenetic analyses were determined with SNP-sites (v2.5.1) [4]. Maximum likelihood phylogenetic tree was inferred using the core SNP alignment with IQ-TREE (v2.1.4) [5-7]. Isolates SRR14745995 to SRR14746025 were not included in the final phylogenetic analyses, as they were identical to the reference strain, *V. alginolyticus* K01M1 (GCA_002119505.2). *V. alginolyticus* strain Vb1394 and reported to carry the previously described *bla*$_{\text{NDM-1}}$ containing-plasmid pC1394 (MH457126.1) were initially isolate from a shrimp sample from a market in Shenzhen, China in 2016 [8]. Yet, no chromosomal genomic data was publicly available for *V. alginolyticus* strain Vb1394. In addition, the plasmid pC1394 (MH457126.1) has not been publicly reported to be carried with any additional bacterial isolates (in both the literature and NCBI nucleotide database searches).

Comparison of the AUSMDU00064140 AMR region

Nucleotide BLAST comparisons were conducted comparing AUSMDU00064140 to the complete genome reference *V. alginolyticus* strain K01M1 (GCA_002119505.2) and to the only reported NDM-1 containing *V. alginolyticus,* strain Vb1394 plasmid pC1394 (MH457126.1) [8].

AUSMDU00064140 Plasmid analyses

The AUSMDU00064140 plasmid, pAUSMDU0064140, was submitted to plasmid PubMLST for typing. The closest incompatibility type match was FII 14.

Mob-suite (v3.0.3 https://pubmed.ncbi.nlm.nih.gov/30052170/) was also used to analyse pAUSMDU00064140. A close match could not be identified. Therefore, nucleotide BLAST comparisons were conducted for pAUSMDU0064140 against the NCBI nt/nr database. The closest match was to the *Vibrio cholerae* strain RFB16 unnamed plasmid (CP043555.1), with a 65% coverage and 73% nucleotide identity.

Data visualisation

The phylogenetic tree was midpoint rooted and visualised with accompanying metatdata in R (v4.1.1) [9] using ggtree (v3.3.0.901) [10-12], ape (v5.5), ggplot2 (v3.3.5) and tidyverse (v1.3.1). Figure 1 plots were combined with aplot (v0.1.1). Nucleotide BLAST comparisons of AUSMDU00064140 to the complete genome reference *V. alginolyticus* strain K01M1 (GCA_002119505.2) and *V. alginolyticus* strain Vb1394 plasmid pC1394 (MH457126.1) were visualized in R using genoPlotR (v0.8.11) [13] and ade4 (v 1.7-18).

Supplementary tables

Supplementary Table 1 Metadata and results of the AMR gene presence summary for all 140 *Vibrio alginolyticus* isolates included in this study.

Supplementary Table 2 Detailed abriTAMR results for *Vibrio alginolyticus* strain AUSMDU00064140.

References

1. Wick, R.R., et al., *Unicycler: Resolving bacterial genome assemblies from short and long sequencing reads.* PLOS Computational Biology, 2017. **13**(6): p. e1005595. 10.1371/journal.pcbi.1005595
2. Feldgarden, M., et al., *AMRFinderPlus and the Reference Gene Catalog facilitate examination of the genomic links among antimicrobial resistance, stress response, and virulence.* Scientific Reports, 2021. **11**(1): p. 12728. 10.1038/s41598-021-91456-0
3. Jolley, K.A., J.E. Bray, and M.C.J. Maiden, *Open-access bacterial population genomics: BIGSdb software, the PubMLST.org website and their applications.* Wellcome Open Res, 2018. **3**: p. 124. 10.12688/wellcomeopenres.14826.1
4. Page, A.J., et al., *SNP-sites: rapid efficient extraction of SNPs from multi-FASTA alignments.* Microbial Genomics, 2016. **2**(4). https://doi.org/10.1099/mgen.0.000056
5. Nguyen, L.-T., et al., *IQ-TREE: A Fast and Effective Stochastic Algorithm for Estimating Maximum-Likelihood Phylogenies.* Molecular Biology and Evolution, 2014. **32**(1): p. 268-274. 10.1093/molbev/msu300
6. Hoang, D.T., et al., *UFBoot2: Improving the Ultrafast Bootstrap Approximation.* Molecular Biology and Evolution, 2017. **35**(2): p. 518-522. 10.1093/molbev/msx281
7. Chernomor, O., A. von Haeseler, and B.Q. Minh, *Terrace Aware Data Structure for Phylogenomic Inference from Supermatrices.* Systematic Biology, 2016. **65**(6): p. 997-1008. 10.1093/sysbio/syw037
8. Zheng, Z., et al., *Identification and Characterization of IncA/C Conjugative, blaNDM-1-Bearing Plasmid in Vibrio alginolyticus of Food Origin.* Antimicrobial Agents and Chemotherapy, 2018. **62**. 10.1128/AAC.01897-18

9. R Core Team, R: A language and environment for statistical computing. R Foundation for Statistical Computing, Vienna, Austria. Journal, 2021. Volume(Issue): p. Pages. https://www.R-project.org/

10. Yu, G., *Using ggtree to Visualize Data on Tree-Like Structures.* Current Protocols in Bioinformatics, 2020. **69**(1): p. e96. https://doi.org/10.1002/cpbi.96

11. Yu, G., et al., *Two Methods for Mapping and Visualizing Associated Data on Phylogeny Using Ggtree.* Molecular Biology and Evolution, 2018. **35**(12): p. 3041-3043. 10.1093/molbev/msy194

12. Yu, G., et al., *ggtree: an r package for visualization and annotation of phylogenetic trees with their covariates and other associated data.* Methods in Ecology and Evolution, 2017. **8**(1): p. 28-36. https://doi.org/10.1111/2041-210X.12628

13. Guy, L., J. Roat Kultima, and S.G.E. Andersson, *genoPlotR: comparative gene and genome visualization in R.* Bioinformatics, 2010. **26**(18): p. 2334-2335. 10.1093/bioinformatics/btq413
