# Supplementary material for: Hidden Resistances: How Routine Whole-Genome Sequencing Uncovered an Otherwise Undetected blaNDM-1 Gene in Vibrio alginolyticus from Imported Seafood
Source: Microbiol Spectr. 2023 Jan 5;11(1):e04176-22. doi: 10.1128/spectrum.04176-22 (PMC9927303; doi:10.1128/spectrum.04176-22)
Supplement: Reviewer comments [file reviewer-comments.pdf]

AMERICAN SOCIETY FOR MICROBIOLOGY | Microbiology Spectrum

Hidden resistances: How routine whole genome sequencing uncovered an otherwise undetected *bla*$_{NDM-1}$ gene in *Vibrio alginolyticus* from imported seafood

Jacqui Morris, Karolina Mercoulia, Mary Valcanis, Claire Gorrie, Norelle Sherry, and Benjamin Howden

Corresponding Author(s): Benjamin Howden, The Peter Doherty Institute for Infection and Immunity

Review Timeline:

Submission Date:	October 13, 2022
Editorial Decision:	November 17, 2022
Revision Received:	December 5, 2022
Accepted:	December 6, 2022

Editor: Cheryl Andam

Reviewer(s): The reviewers have opted to remain anonymous.

Transaction Report:

(Note: With the exception of the correction of typographical or spelling errors that could be a source of ambiguity, letters and reports are not edited. The original formatting of letters and referee reports may not be reflected in this compilation.)

DOI: https://doi.org/10.1128/spectrum.04176-22

November 17, 2022

Prof. Benjamin P Howden
Doherty Institute
MDU Public Health Laboratory, Level 1
792 Elizabeth St
Melbourne, Victoria 3000
Australia

Re: Spectrum04176-22 (Hidden resistances: How routine whole genome sequencing uncovered an otherwise undetected bla_{NDM-1} gene in *Vibrio alginolyticus* from imported seafood)

Dear Prof. Benjamin P Howden:

Thank you for submitting your manuscript to Microbiology Spectrum. When submitting the revised version of your paper, please provide (1) point-by-point responses to the issues raised by the reviewers as file type "Response to Reviewers," not in your cover letter, and (2) a PDF file that indicates the changes from the original submission (by highlighting or underlining the changes) as file type "Marked Up Manuscript - For Review Only". Please use this link to submit your revised manuscript - we strongly recommend that you submit your paper within the next 60 days or reach out to me. Detailed instructions on submitting your revised paper are below.

Link Not Available

Below you will find instructions from the Microbiology Spectrum editorial office and comments generated during the review.

ASM policy requires that data be available to the public upon online posting of the article, so please verify all links to sequence records, if present, and make sure that each number retrieves the full record of the data. If a new accession number is not linked or a link is broken, provide production staff with the correct URL for the record. If the accession numbers for new data are not publicly accessible before the expected online posting of the article, publication of your article may be delayed; please contact the ASM production staff immediately with the expected release date.

The ASM Journals program strives for constant improvement in our submission and publication process. Please tell us how we can improve your experience by taking this quick Author Survey.

Sincerely,

Cheryl Andam

Editor, Microbiology Spectrum

Journals Department
American Society for Microbiology
1752 N St., NW
Washington, DC 20036
E-mail: spectrum@asmusa.org

Reviewer comments:

Reviewer #1 (Comments for the Author):

Review
The authors describe a new V. alginolyticus strain isolated from cooked prawn imported from Thailand. This strain was found to possess a gene associated with resistance to carbapenems class of antibiotics, but it was unique in that 1/ this gene was located on the largest chromosome rather than on a plasmid, and 2/ the strain still appeared to be sensitive to carbapenem.
This is a well-written manuscript and the methods and conclusions seem sound. I only have a few suggestions listed below.

1. It seems that NDM was not spelled out but it may be helpful to do so for readers that may not be familiar with this acronym. Similarly, defining blaNDM-1 gene would be helpful.

2. The V. alginolyticus strain studied here was isolated from cooked prawns and it is difficult to understand how a viable strain could be isolated from cooked food. Please add an explanation or possible explanations for this.

3. It is mentioned that routine screening is only targeting V. cholerae so it would be important to add more information regarding the context in which V. alginolyticus (and V. fluvialis) were isolated from the cooked prawns including the isolation method. Please add such clarifications.

Reviewer #2 (Comments for the Author):

This manuscript is a brief report of the identification of a Vibrio alginolyticus strain carrying a blaNDM gene giving resistance to beta lactic antibiotics. This is a gene rarely found in vibrios and certainly worthy of reporting to the community, alerting us of a potential rising threat of AMR in aquaculture associated bacteria.

The paper analyzes one genome sequence in details. Its does a good job of framing the context and importance of the find.

I just have a few minor comments:
-There needs to be a bit more discussion about plasmid pC1349 and the strains carrying it in relation to its origin (China) and the origin of the imported shrimps from which the blaNDM V, alginolyticus strains isolated in this study
- The tree presented in figure 1 needs a higher resolution, as the branch support values are not readable

Staff Comments:

Preparing Revision Guidelines
To submit your modified manuscript, log onto the eJP submission site at https://spectrum.msubmit.net/cgi-bin/main.plex. Go to Author Tasks and click the appropriate manuscript title to begin the revision process. The information that you entered when you first submitted the paper will be displayed. Please update the information as necessary. Here are a few examples of required updates that authors must address:

• Point-by-point responses to the issues raised by the reviewers in a file named "Response to Reviewers," NOT IN YOUR COVER LETTER.
• Upload a compare copy of the manuscript (without figures) as a "Marked-Up Manuscript" file.
• Each figure must be uploaded as a separate file, and any multipanel figures must be assembled into one file.
• Manuscript: A .DOC version of the revised manuscript
• Figures: Editable, high-resolution, individual figure files are required at revision, TIFF or EPS files are preferred

For complete guidelines on revision requirements, please see the journal Submission and Review Process requirements at https://journals.asm.org/journal/Spectrum/submission-review-process. **Submissions of a paper that does not conform to Microbiology Spectrum guidelines will delay acceptance of your manuscript.** "

Please return the manuscript within 60 days; if you cannot complete the modification within this time period, please contact me. If you do not wish to modify the manuscript and prefer to submit it to another journal, please notify me of your decision immediately so that the manuscript may be formally withdrawn from consideration by Microbiology Spectrum.

If your manuscript is accepted for publication, you will be contacted separately about payment when the proofs are issued; please follow the instructions in that e-mail. Arrangements for payment must be made before your article is published. For a complete list of **Publication Fees**, including supplemental material costs, please visit ourwebsite.

Corresponding authors may join or renew ASM membership to obtain discounts on publication fees. Need to upgrade your membership level? Please contact Customer Service at Service@asmusa.org.

Thank you for submitting your paper to Microbiology Spectrum.

Reviewer	Comments/ Suggestions for Authors	Authors response
1	1. It seems that NDM was not spelled out but it may be helpful to do so for readers that may not be familiar with this acronym. Similarly, defining blaNDM-1 gene would be helpful.	We have provided additional information on lines 39-41 spelling out NDM and noted the first description of blaNDM-1.
	2. The V. alginolyticus strain studied here was isolated from cooked prawns and it is difficult to understand how a viable strain could be isolated from cooked food. Please add an explanation or possible explanations for this.	We have provided additional information on lines 159-163. Including cooking time for prawns is short and if prawns are densely packed during the boiling process, they will not cook uniformly. Viable bacteria may remain but also be introduced after cooking. Cross contamination during the production process is an issue if prawns are not immediately frozen or processed for sale. Both factors may lead to contaminated product sold as a cooked product ready for consumption.
	3. It is mentioned that routine screening is only targeting V. cholerae so it would be important to add more information regarding the context in which V. alginolyticus (and V. fluvialis) were isolated from the cooked prawns including the isolation method. Please add such clarifications.	We have included additional information on lines 154-156. Food testing laboratories generally do not have capacity to ID *Vibrio* species. Therefore, typical colonies are referred to a reference laboratory (ie. Microbial Diagnostic Unit Public Health Laboratory, MDU PHL) for ID and characterisation. Both *V. alginolyticus* and *V. fluvialis* grow as large yellow colonies on selective TCBS agar and resemble *V. cholorae*. It is also routine practice to store, sequence and screen all *Vibrio* isolates for AMR determinants at MDU PHL regardless of requested testing.
2	There needs to be a bit more discussion about plasmid pC1349 and the strains carrying it in relation to its origin (China) and the origin of the imported shrimps from which the blaNDM V, alginolyticus strains isolated in this study	We have provided more information in the Figure 1 legend and in the supplementary information on lines 364-369 on *V. alginolyticus* strain Vb1394, carrying the previously described *bla*$_{NDM-1}$ containing-plasmid pC1394 (MH457126.1). This includes information on its initial

		source and that no chromosomal or genomic data is publicly available for the *V. alginolyticus* strain. In addition, the plasmid pC1394 has not been publicly reported with any additional bacterial isolates to date. The authors do not have additional information on the source of the sample, other than the Country of origin.
	The tree presented in figure 1 needs a higher resolution, as the branch support values are not readable	We have updated the font size and quality of Figure 1.
Authors Addition		We have updated some small typos (which are also highlighted in the Marked-up Manuscript) an also included the SRA dataset IDs and the genome accession reference IDs that are now available, in Supplementary Table 1 and manuscript where relevant.

December 6, 2022

Prof. Benjamin P Howden
The Peter Doherty Institute for Infection and Immunity
MDU Public Health Laboratory, Level 1
792 Elizabeth St
Melbourne, Victoria 3000
Australia

Re: Spectrum04176-22R1 (Hidden resistances: How routine whole genome sequencing uncovered an otherwise undetected *bla*$_{NDM-1}$ gene in *Vibrio alginolyticus* from imported seafood)

Dear Prof. Benjamin P Howden:

Your manuscript has been accepted, and I am forwarding it to the ASM Journals Department for publication. You will be notified when your proofs are ready to be viewed.

The ASM Journals program strives for constant improvement in our submission and publication process. Please tell us how we can improve your experience by taking this quick Author Survey.

As an open-access publication, Spectrum receives no financial support from paid subscriptions and depends on authors' prompt payment of publication fees as soon as their articles are accepted. You will be contacted separately about payment when the proofs are issued; please follow the instructions in that e-mail. Arrangements for payment must be made before your article is published. For a complete list of **Publication Fees**, including supplemental material costs, please visit ourwebsite.

ASM policy requires that data be available to the public upon online posting of the article, so please verify all links to sequence records, if present, and make sure that each number retrieves the full record of the data. If a new accession number is not linked or a link is broken, provide production staff with the correct URL for the record. If the accession numbers for new data are not publicly accessible before the expected online posting of the article, publication of your article may be delayed; please contact the ASM production staff immediately with the expected release date.

Corresponding authors may join or renew ASM membership to obtain discounts on publication fees. Need to upgrade your membership level? Please contact Customer Service at Service@asmusa.org.

Thank you for submitting your paper to Spectrum.

Sincerely,

Cheryl Andam
Editor, Microbiology Spectrum

Journals Department
American Society for Microbiology
1752 N St., NW
Washington, DC 20036
E-mail: spectrum@asmusa.org

Supplemental Material: Accept
Supplemental Table 2: Accept
Supplemental Material: Accept
